# 

**Published:** 1919

**Authors:** 


### NINETY-SEVENTH LIST OF BOOKS.

The following books have been received since the publication of the list in December, 1916 :—

*25th March*, 1919.

| | | | |
|---|---|---|---|
| **Anders, J. M.** | *Practice of Medicine*, 2 vols. | 4th Ed. | 1900 |
| **Atkinson, W. B.** | *Therapeutics of Gynæcology of Obstetrics* | 2nd Ed. | 1881 |
| **Atthill, L.** | *Diseases of Women* | 6th Ed. | 1880 |
| **Auerbach, S.** | *Headache* (Trans. by E. Playfair) | | 1913 |
| **Ball, Sir C. B.** | *Diseases of the Rectum* | 2nd Ed. | 1910 |
| **Ballenger and A. Wippern, W.** | *Diseases of Eye, Ear, Nose and Throat* | | 1902 |
| **Barcroft, J.** | *Respiratory Function of the Blood* | | 1914 |
| **Bastedo, W. A.** | *Materia Medica and Pharmacology* | | 1913 |
| **Basu, B. D.** | *Dietetic Treatment of Diabetes* | 9th Ed. | 1918 |
| **Bayliss, W. M.** | *Principles of General Physiology* | 2nd Ed. | 1918 |
| **Bennet, W.** | *Toxicological Chart* | | 1826 |
| **Bennett, R. R.** | *Medical and Pharmaceutical Latin* | | 1906 |

| | | | |
|---|---|---|---|
| **Binet and T. Simon, A.** | *Mentally Defective Children* (Trans. by W. B. Drummond) | | [1914] |
| **Bristowe, W. R.** | *Treatment of Joint and Muscle Injuries* | | 1917 |
| **Browning, C. H.** | *Applied Bacteriology* | | 1918 |
| **Browning and I. McKenzie, C. H.** | *Recent Methods in Diagnosis of Syphilis* | | 1912 |
| **Broca, A.** | *Ligations and Amputations* (Trans. by E. Ward) | | 1917 |
| **Buchanan, R. J. M.** | *The Blood in Health and Disease* | | 1909 |
| **Buckmaster, G. A.** | *The Morphology of Blood* | | 1906 |
| **Burkett, W. C.** | *Bibliography of W. C. Welch* | | 1917 |
| **Caird and C. W. Cathcart, F. M.** | *Surgical Handbook* | 15h Ed. | 1910 |
| **Campbell, H.** | *Aids to Pathology* | 3rd Ed. | 1915 |
| **Campbell, H. J.** | *Elementary Biology* | 2nd Ed. | 1895 |
| **Carrel and G. Dehelly, A.** | *Treatment of Infected Wounds* (Trans. by H. Child) | 2nd Ed. | 1918 |
| **Carter and W. A. Frost, R. B.** | *Ophthalmic Surgery* | | 1887 |
| *Catalogue (Index-) of the Library of the Surgeon-General's Office, U.S.A.*, 2nd Series, Vols. XIX.—XXI. | | | 1914–16 |
| **Chaplin, H. D.** | *Infant Feeding* | | 1903 |
| **Chaucer, G.** | *The Prologue to the Canterbury Tales* (Ed. by W. W. Skeat) | 3rd Ed. | 1900 |
| **Cheadle and F. J. Poynton, W. B.** | *Artificial Feeding of Infants* | 6th Ed. | 1906 |
| **Cohen, J. B.** | *Organic Chemistry* | | [1912] |
| **Colbeck, E. H.** | *Diseases of the Heart* | 2nd Ed. | 1905 |
| **Corbin and A. M. Stewart, H. E.** | *Handbook of Chemistry and Physics* | 4th Ed. | 1911 |
| **Cripps, R. A.** | *Galenic Pharmacy* | | 1893 |
| **Cushny, A. R.** | *Pharmacology and Therapeutics* | 5th Ed. | 1910 |
| *Cyclopædia of English Literature*, 2 vols. | | | 1858–60 |
| **Da Costa, J. C.** | *Modern Surgery* | 4th Ed. | 1904 |
| **Daw, S. W.** | *Orthopædic Effects of Gunshot Wounds* | | 1919 |
| **Dawson, E. R.** | *The Causation of Sex in Man* | 2nd Ed. | 1917 |
| **Daniell, A.** | *Principles of Physics* | | 1884 |
| **Davies, T.** | *Preparation of Microscopic Objects* | 2nd Ed. | 1873 |
| **Dawson, Sir B.** | *The Nation's Welare* | | 1918 |
| **Donald, A.** | *Introduction to Midwifery* | 5th Ed. | 1902 |
| **Dorland, W. A. N.** | *Illustrated Medical Dictionary* | 7th Ed. | 1913 |
| **Dunman, T.** | *Electricity and Magnetism* | | 1888 |
| **Eder, M. D.** | *War Shock* | | 1917 |
| **Elliott, R. H.** | *Glaucoma : Handbook for General Practitioners* | | 1917 |
| ,, | *Glaucoma : A Handbook for Students* | | 1918 |
| ,, | *The Indian Operation for Couching for Cataract* | | 1917 |
| **Emery, W. D.** | *Clinical Bacteriology* | 5th Ed. | 1917 |
| *Encyclopædia Medica*, Vol. I.—V. | | 2nd Ed. | 1915–17 |
| **Faught, F. A.** | *Blood Pressure* | | 1913 |
| **Fiske, A.** | *Structure amd Functions of the Body* | | 1911 |
| **Fothergill, J. M.** | *Aids to Rational Therapeutics* | | 1881 |

| | | |
|---|---|---|
| **French, H.** [Ed.] .. | *Index of Differential Diagnosis* .. 2nd Ed. | 1917 |
| **Friel, A. R.** .. .. | *Obiter Scripta : Throat, Nose and Ear* .. .. | 1914 |
| **Fuchs, L.** .. .. | *De medendi methodo libri quatuor* .. .. .. | 1539 |
| **Furneaux, W. S.** .. | *Elementary Chemistry* .. .. .. .. .. .. | 1890 |
| **Gadd, H. W.** .. .. | *The Poisons and Pharmacy Act,* 1908 .. .. | 1909 |
| **Garson and C. H. Read, J. G.** | *Notes and Queries on Anthropology* 2nd Ed. | 1892 |
| **Garton, W.** .. .. | *Electro-Therapeutics* .. .. .. .. .. .. .. | 1917 |
| **Gay, F. P.** .. .. | *Typhoid Fever* .. .. .. .. .. .. .. .. | 1918 |
| **Godlee, Sir R. J.** .. | *Lord Lister* .. .. .. .. .. .. .. .. | 1917 |
| **Gould, Sir A. P. and E. P.** | *Surgical Diagnosis*.. .. .. 5th Ed. | 1919 |
| **Gray, H.** .. .. | *Anatomy* (Ed. by R. Howden) ..20th Ed. | 1918 |
| **Greenish, H. G.** .. | *Introduction to Materia Medica* .. .. .. | 1899 |
| **Greenwood, W. O.** | *Scopolamine-Morphine* .. .. .. .. .. | 1918 |
| **Hampson, W.** .. | *Radium Explained* .. .. .. .. .. .. .. | 1905 |
| **Harrison, L. W.** .. | *Venereal Diseases* .. .. .. .. .. .. .. | 1918 |
| **Hayes, R.** .. .. | *Intensive Treatment of Syphilis* .. .. .. | 1917 |
| **Hebblethwaite, S. M.** | *Notes on General Practice* .. .. .. .. | 1905 |
| **Hektoen and D. Riesmann, L.** | *Text-Book of Pathology,* 2 vols. .. | 1901 |
| **Hewer, Mrs. J. L.** .. | *Our Baby* .. .. .. .. .. .. ..16th Ed. | 1918 |
| **Hewlett, R. T.** .. | *Pathology* .. .. .. .. .. .. .. .. .. | 1906 |
| **Hinshelwood, J.** .. | *Congenital Word Blindness* .. .. .. .. .. | 1917 |
| **Hoblyn, R. D.** .. | *Dictionary of Terms* (Ed. by J. A. Price) 12th Ed. | 1892 |
| **Hoffman, F. L.** .. | *Mortality from Cancer throughout the World* .. | 1915 |
| **Holden, L.** .. .. | *Human Osteology* (Ed. by C. Stewart) 8th Ed. | 1899 |
| **Holt, L. E.** .. .. | *Diseases of Infancy and Childhood* .. .. .. | 1900 |
| **Huggins, G. M.** .. | *Amputation Stumps : Care and Treatment* .. | 1918 |
| **Humphrey, J.** .. | *Materia Medica* .. .. .. .. .. 2nd Ed. | 1907 |
| **Hurst, A. F.** .. .. | *Medical Diseases of the War* .. .. .. .. | 1916 |
| **Hyde and F. H. Montgomery, J. N.** | *Diseases of the Skin* .. 5th Ed. | 1900 |
| *Infectious Diseases in Schools, Rules for Preventing* .. | .. .. .. .. | 1916 |
| **Jago, W.** .. .. | *Inorganic Chemistry* .. .. .. .. .. 2nd Ed. | 1890 |
| **James, W.** .. .. | *Psychology* .. .. .. .. .. .. .. .. .. | [1892] |
| **Johnston, J. F. W.** | *The Chemistry of Common Life,* 2 vols. .. .. | 1854–55 |
| **Jones and L. J. Llewellyn, A. B.** | *Malingering* .. .. .. .. .. .. | 1917 |
| **Jones, R.** .. .. | *Notes on Military Orthopædics* .. .. .. | 1917 |
| **Jordan, E. O.** .. | *General Bacteriology* .. .. .. .. 6th Ed. | 1918 |
| **Keith, A.** .. .. | *The Human Body* .. .. .. .. .. .. .. | [N.D.] |
| **Kidd, F.** .. .. | *Common Diseases of the Male Urethra* .. .. | 1917 |
| **Knox, R.** .. .. | *Radiography and Radio-Therapeutics.* Vol. I. | 1917 |
| **Lamb, W.** .. .. | *Diseases of the Nose, Throat and Ear* 4th Ed. | 1917 |
| **Lane, Sir A.** .. .. | *Operative Treatment of Chronic Intestinal Stasis* .. .. .. .. .. .. .. 4th Ed. | 1918 |
| **Lewis and A. Balfour, C. J.** | *Public Health* .. .. .. .. .. .. | 1902 |
| **Lownds, L.** .. .. | *First Book of Physics* .. .. .. .. .. .. | 1910 |
| **Lucas, E. W.** .. | *Practical Pharmacy* .. .. .. .. .. .. .. | 1898 |
| **McDonald, D. M.** .. | *Practical Prescribing and Treatment* .. .. | 1915 |

| | | | |
|---|---|---|---|
| **McGarrison, R.** .. | *The Thyroid Gland in Health and Disease* .. | | 1917 |
| **McKendrick, A.** .. | *Back Injuries* .. .. .. .. .. .. .. .. | | 1916 |
| **MacKenzie, W. C.** | *The Action of Muscles* .. .. .. .. .. .. | | 1918 |
| **Macleod, J. M. H.** | *Burns and their Treatment* .. .. .. .. .. | | 1918 |
| **Malley, A. C.** .. | *Photo-Micrography* .. .. .. .. .. | 2nd Ed. | 1885 |
| **Manson, Sir P.** .. | *Tropical Diseases* .. .. .. .. .. | 6th Ed. | 1917 |
| **Marshall, C. R.** .. | *Manual of Prescribing* .. .. .. .. .. .. | | 1908 |
| **Martin, S.** .. .. | *Manual of General Pathology* .. .. .. .. | | 1904 |
| **Maudsley, H.** .. | *Responsibility in Mental Disease* .. .. .. .. | | 1874 |
| **Maxwell, W. H.** .. | *Ventilation, Heating and Lighting* .. | 2nd Ed. | 1907 |
| **May, O.** .. .. .. | *The Prevention of Venereal Diseases* .. .. | | 1918 |
| **Mercier, C. A.** .. | *Crime and Insanity* .. .. .. .. .. .. .. .. | | [N.D]. |
| **Morison, Sir A.** .. | *Insanity* .. .. .. .. .. .. .. .. | 4th Ed. | 1848 |
| **Morison, R.** .. .. | *Bipp Treatment of War Wounds* .. .. .. | | 1918 |
| **Morris, E. W.** .. | *History of the London Hospital* .. .. .. | | 1910 |
| **Morris, Sir M.** .. | *The Nation's Health* .. .. .. .. .. .. .. .. | | 1917 |
| ,, .. | *Diseases of the Skin* .. .. .. .. .. | 6th Ed. | 1917 |
| **Muir and J. Ritchie, R.** | *Manual of Bacteriology* .. .. | 7th Ed. | 1919 |
| **Muirhead, I. B.** .. | *Extra-Ocular Pressure and Myopia* .. .. | | 1916 |
| **Myers, C. S.** .. .. | *Introduction to Experimental Psychology* .. | | 1912 |
| **Newth, G. S.** .. .. | *Inorganic Chemistry* .. .. .. .. .. | 12th Ed. | 1907 |
| ,, .. .. | *Chemical Lecture Experiments* .. | 2nd Ed. | 1908 |
| ,, .. .. | *Elementary Practical Chemistry* .. .. .. | | 1904 |
| **Nicoll, M.** .. .. | *Dream Psychology* .. .. .. .. .. .. .. .. | | 1917 |
| **Noble, D.** .. .. | *The Human Mind* .. .. .. .. .. .. .. .. | | 1858 |
| **Nunn, A. W.** .. | *Materia Medica* .. .. .. .. .. .. .. .. | | 1911 |
| **Oliver, J. W.** .. .. | *Elementary Botany* .. .. .. .. .. | 17th Ed. | 1908 |
| **Page, C. M.** .. .. | *Medical Field Service Hand-book* .. .. .. | | 1919 |
| **Parkes and H. R. Kenwood, L. C.** | *Hygiene and Public Health* | 6th Ed. | 1917 |
| **Parsons, H. F.** .. | *Isolation Hospitals* .. .. .. .. .. .. .. .. | | 1914 |
| **Paterson, A. M.** .. | *Anatomy of Peripheral Nerves* .. .. .. | | 1919 |
| *Pathology* (*Catechism Series*). Part IV. | .. .. .. .. .. | 2nd Ed. | 1917 |
| **Penhallow, D. P.** .. | *Military Surgery* .. .. .. .. .. | 2nd Ed. | 1918 |
| **Pickering, C.** .. | *The Races of Man* .. .. .. .. .. | New Ed. | 1850 |
| **Pilley, J. J.** .. .. | *Hygiene* .. .. .. .. .. .. .. .. | 3rd Ed. | 1888 |
| **Porter, C.** .. .. | *Hygiene and Public Health* .. .. .. .. .. | | 1917 |
| **Power, D'A.** .. .. | *On Cancer of the Tongue* .. .. .. .. .. .. | | 1919 |
| **Poyser, A. W.** .. | *Magnetism and Electricity* .. .. | 10th Ed. | 1903 |
| **Price, F. W.** .. .. | *Diseases of the Heart* .. .. .. .. .. .. | | 1918 |
| **Price-Jones, C.** .. | *Blood Pictures* .. .. .. .. .. .. .. .. .. | | 1917 |
| **Proctor, B. S.** .. | *Practical Pharmacy* .. .. .. .. .. | 3rd Ed. | 1892 |
| **Pye, [W.]** .. .. | *Surgical Handicraft* (Ed. by W. H. Clayton-Green) .. .. .. .. .. .. .. .. | 8th Ed. | 1919 |
| **Ramsbotham, F. H.** | *Obstetric Medicine and Surgery* .. | 5th Ed. | 1867 |
| **Reinhardt, C.**.. .. | *Mental Therapeutics* .. .. .. | 3rd Ed. | 1914 |
| **Remsen, I.** .. .. | *Organic Chemistry* .. .. .. .. .. | 3rd Ed. | 1899 |
| ,, .. .. | *Elements of Chemistry* .. .. .. .. .. .. | | 1905 |
| *Research Defence Society, Publications of the* | .. .. .. .. .. .. | | 1909 |

| | | | |
|---|---|---|---|
| **Riverius, L.** | *The Compleat Practice of Physick* | | 1655 |
| **Riviere, C.** | *Pneumothorax Treatment of Pulmonary Tuberculosis* | | 1917 |
| **Roberts, W.** | *Lectures on Dietetics and Dyspepsia* | | 1885 |
| **Roberts, W. G. A.** | *Clinical Diagnosis* | | 1896 |
| **Royle, J. F.** | *Materia Medica* (Ed. by J. Harvey) | 6th Ed. | 1876 |
| **Roscoe and C. Schorlemmer, H. E.** | *Treatise on Chemistry.* Vols. I; II., Pt. I.; III., Pt. I. | | 1880–81 |
| **Sankey, W. H. O.** | *Mental Diseases* | | 1866 |
| **Savage, W. G.** | *Bacteriological Examination of Food and Water* | | 1914 |
| **Sachs, J.** | *Text-Book of Botany* | 2nd Ed. | 1882 |
| **Schafer, E. A.** | *Experimental Physiology* | New Ed. | 1916 |
| **Schmiedeberg, O.** | *Elements of Pharmacology* | | 1887 |
| **Semple, C. E. A.** | *Materia Medica and Therapeutics* | | 1892 |
| **Shaw, T. C.** | *Ex Cathedrâ Essays on Insanity* | | 1904 |
| **Short, A. R.** [Ed.] | *Index of Prognosis* | 2nd Ed. | 1918 |
| **Simon, W.** | *Manual of Chemistry* | 8th Ed. | 1905 |
| **Smith, J. L.** | *Diseases of Infancy and Childhood* | 5th Ed. | 1881 |
| **Squire, P. W.** | *Pocket Companion to the P.B.* | 2nd Ed. | 1908 |
| ,, | *Companion to the B.P.* | 18th Ed. | 1908 |
| **Stark, A. C.** | *Aids to Practical Pharmacy* | | 1898 |
| **Stewart and E. S. Lawrence, D.D.** | *Essentials of Medical Electricity* | | 1893 |
| **Stewart, R. W.** | *Magnetism and Electricity* | 3rd Ed. | 1898 |
| **Stewart, Sir T. G.** | *Lectures on Giddiness, etc.* | 2nd Ed. | 1898 |
| **Sutton, E.** | *Naval Hospital Ship* | | 1918 |
| **Sutton, J. B.** | *Tumours: Innocent and Malignant* | 6th Ed. | 1917 |
| **Taine, H.** | *On Intelligence* (Trans. by T. D. Haye) | | 1871 |
| **Tanzi, E.** | *Text-Book of Mental Diseases* (Translated) | | 1909 |
| **Taylor, J.** | *Paralysis and other Diseases of Nervous System* | | 1905 |
| **Theobald, S.** | *Prevalent Diseases of the Eye* | | 1907 |
| **Thompson, C. J. S.** | *Compendium of Pharmacopœias* | 4th Ed. | 1914 |
| **Thompson, M.** | *The Apothecaries' Hall Manual* | | 1903 |
| **Thorne, L. T.** | *The "Nauheim" Treatment* | 5th Ed. | 1918 |
| **Thornton, E. G.** | *Dose-Book and Manual of Prescription Writing* | 2nd Ed. | 1901 |
| **Thorpe and M. Muir, T. E.** | *Qualitative Chemical Analysis* | 2nd Ed. | 1878 |
| **Topinard, P.** | *Anthropology* (Translated) | | 1894 |
| **Treves, Sir F.** | *Surgical Applied Anatomy* (Ed. by A. Keith and W. C. Mackenzie) | 7th Ed. | 1918 |
| **Underhill, F. P.** | *Physiology of the Amino Acids* | | 1915 |
| **Upcott, H.** | *Antiseptic Methods* | | 1907 |
| **Walford, W. G.** | *Dangers in Neck Wear* | | 1917 |
| **Walker, C. E.** | *Hereditary Characters and their Mode of Transmission* | | 1910 |
| **White and J. Humphreys, E.** | *Pharmacopœdia* | 2nd Ed. | 1909 |
| **Watson, C.** | *Lectures on Medicine for Nurses* | | 1917 |
| **Whiteford, C. H.** | *Acute Appendicitis* | | 1917 |

| | | |
|---|---|---|
| **Whitla, Sir W.** | *Theory and Practice of Medicine*, 2 vols. | 1908 |
| **Wills, G. S. V.** | *Manual of Practical Analysis* .. .. 11th Ed. | [N.D.] |
| ,, | *Elements of Pharmacy* .. .. .. 9th Ed. | 1908 |
| **Winckel, F.** | *Diseases of Women* (Trans. by F. Parvin) .. 2nd Ed. | 1890 |
| **Wise, T. A.** | *Hindu System of Medicine* | 1845 |
| **Whittaker, C. R.** | *Surgery* (*Catechism Series*). Part V. 3rd Ed. | 1917 |
| **Wateson, [G.]** | *The Cures of the Diseased* [Reprint]. (Ed. by C. Singer) | 1915 |
| **Webb-Johnson, A. E.** | *Surgical Aspects of Typhoid and Para-Typhoid Fever* | 1919 |
| **White, W. H.** | *Materia Medica* .. .. .. .. 16th Ed. | 1918 |
| **Wilson, R. M.** | *The Hearts of Man* | 1918 |
| **Wingfield, H.** | *Forms of Alcoholism and their Treatment* | 1919 |
| **Winslow, F.** | *The Anatomy of Suicide* | 1840 |
| **Wood, R. C.** | *The Soldier's First Aid* | 1917 |
| **Yealland, L. R.** | *Hysterical Disorders of Warfare* | 1918 |
| **Younger, E. G.** | *Insanity in Everyday Practice* .. 4th Ed. | 1917 |

### TRANSACTIONS, REPORTS, JOURNALS, etc.

American Association of Obstetricians and Gynecologists, Transactions of the .. .. .. .. .. .. Vols. XXVII.—XXX. 1914–17

American Journal of Obstetrics .. .. Vols. LXX.—LXXIII. 1915–16

American Journal of Ophthalmology, The .. .. .. Vol. XXXII. 1915

American Journal of Surgery, The .. .. .. .. .. .. .. .. 1914–15

American Journal of the Medical Sciences .. Vols. CXLVIII—CL. 1914–16

American Laryngological Association, Transactions of the.. .. .. 1914–17

American Laryngological, Rhinological and Otological Society, Transactions of the.. .. .. .. .. .. .. .. .. .. .. .. 1915–17

American Ophthalmological Society, Transactions of the .. 1915–18

American Pediatric Society, Transactions of the Vols. XXVI.—XXVIII. 1914–16

American Surgical Association, Transactions of the Vols. XXXII.—XXXV. 1914–17

Boston Medical and Surgical Journal, The Vols. CLXXI.—CLXXIV. 1914–16

Brain, Vols. IX.—XXXIX, 1887–1917; Index Vols. I.—XXIII. .. 1902

Bristol Health Report .. .. .. .. .. .. .. .. .. .. .. .. .. .. 1916

Bristol Medico-Chirurgical Journal, The Vols. XXXII., XXXIII. 1914–15

British Journal of Dermatology .. .. .. .. .. .. .. .. .. 1914–15

British Journal of Tuberculosis .. .. .. .. .. Vols. VIII., IX. 1914–15

British Medical Journal, The .. .. .. Vol. II., 1914—Vol. II., 1918

Bulletin de l'Académie de Médecine .. .. .. .. .. .. .. .. 1914–15

Canadian Medical Association Journal .. .. Vols. IV., V. 1914–15

College of Physicians of Philadelphia, Transactions of the Vols. XXXVI.—XXXIX. 1914–17

Edinburgh Medical Journal, The .. .. N.S., Vols. XIII.—XVI. 1914–16

| | | |
|---|---|---|
| English Catalogue of Books for 1915 .. .. .. .. .. .. .. .. | | 1916 |
| Glasgow Medical Journal .. .. | Vols. LXXXII.—LXXXV. | 1914–15 |
| Hospitals— | | |
| Episcopal Hospital Reports .. .. .. .. | Vols. III., IV. | 1915–16 |
| Guy's Hospital Reports .. .. .. | Vols. LXVIII., LXIX. | 1914–18 |
| Johns Hopkins Hospital Reports .. .. .. .. | Vol. XVII. | 1916 |
| St. Bartholomew's Hospital Reports .. .. | Vols. LI., LII. | 1915–16 |
| St. Thomas's Hospital Reports .. .. | Vols. XLII.—XLIV. | 1913–18 |
| Indian Medical Gazette .. .. .. .. .. .. .. | Vols. XLIX., L. | 1914–15 |
| International Abstract of Surgery.. .. .. | Vols. XX.—XXII. | 1915–16 |
| Ireland, Transactions of the Royal Academy of Medicine in | Vol. XXXV. | 1917 |
| Jahrbericht über die Neurologie und Psychiatrie .. .. .. | | 1899–1912 |
| Johns Hopkins Hospital Bulletin .. .. | Vols. XXV., XXVI. | 1914–15 |
| Journal of Experimental Medicine .. .. .. .. | Vol. XX. | 1914 |
| Journal of Laryngology .. .. .. .. | Vols. XXIX., XXX. | 1914–15 |
| Journal of Medical Research .. .. .. .. .. | Vol. XXXIV. | 1916 |
| Journal of Mental Science .. .. .. .. .. .. | Vol. LXI. | 1915 |
| Journal of Tropical Medicine .. .. .. .. .. | Vol. XVIII. | 1915 |
| Lancet, The .. .. .. .. .. .. .. | Vols. II., 1914—Vol. II. | 1918 |
| London Dermatological Society, Transactions of the.. .. .. | | 1914–19 |
| Medical Annual, The .. .. .. .. .. .. .. .. .. | | 1915–18 |
| Medical Officer, The.. .. .. .. .. .. .. | Vols. XII.—XV. | 1915–16 |
| Medical Press and Circular, The .... .. .. .. | Vol. CLII. | 1916 |
| Medical Record.. .. .. .. | Vols. LXXXVIII., LXXXIX. | 1915–16 |
| Medical Review .. .. .. .. .. .. .. .. .. | Vol. XVIII. | 1915 |
| Medical Society of London, Transactions of the | Vols. XXXVII.—XL. | 1914–17 |
| New York Obstetrical Society, Transactions of the .. .. .. | | 1911–13 |
| New Zealand Medical Directory, The .. .. .. .. .. .. .. .. | | 1915 |
| Ophthalmological Transactions .. | Vols. XXXV.—XXXVIII. | 1915–18 |
| Parasitology .. .. .. .. .. .. .. .. .. .. .. | Vol. VII. | 1914 |
| Pharmaceutical Society, Calendar of the .. .. .. .. .. .. | | 1916–17 |
| Philippine Journal of Science, Index to Vols. I.—X. .. .. .. | | 1917 |
| Practitioner, The .. .. .. .. .. .. | Vols. XCIII.—XCVI. | 1914–16 |
| Quarterly Journal of Medicine .. .. .. | Vols. VII., VIII. | 1914–15 |
| Royal College of Physicians of London, List of Fellows, etc. .. .. | | 1917 |
| Royal Society of Medicine, Proceedings of the | Vols. VII., VIII. | 1914–15 |
| Smithsonian Report.. .. .. .. .. .. .. .. .. .. | | 1914–17 |
| Studies from the Department of Pathology, Columbia University | Vols. XV., XVI. | 1916–17 |
| Surgery, Gynecology and Obstetrics .. .. | Vols. XXII., XXIII. | 1915–16 |
| Therapeutic Gazette .. .. .. .. .. .. .. | Vol. XXXIX. | 1915 |
| West London Medical Journal .. .. .. .. .. | Vol. XX | 1915 |
| Year-Book of Scientific and Learned Societies .. .. .. .. .. | | 1917 |

The subscription to Lewis's Medical and Scientific Library has been resumed. The Hon. Librarian will be pleased to obtain from that source any books desired by members of the Medico-Chirurgical Society.

### Local Medical Notes.

**University of Bristol.**—The undermentioned students have recently passed the examinations noted :—

M.B., Ch.B.—*Second Examination, Part I.* : F. H. Bodman, J. R. Duerden, J. M. Evans, J. E. S. Hamilton, F. J. Hector, Marguerite G. Hughes, Frances M. Jones, Muriel V. Joscelyne, A. J. Keevil, E. C. K. Kenderdine, F. Langford, Carrie H. Osmond, Doris M. Pullen, J. A. L. Roberts, Dorothy Staley, I. Williams. *Part II.:* Phyllis Beames, A. W. Fawcett, Madge E. Golding, Winifred G. Nott, P. Phillips, J. A. Prichard, Hilda E. Reynolds, Victoria S. Tryon.

*Final Examination, Part II.:* Evelyn B. Salter, Elizabeth Casson, A. D. Symons, A. G. Bodman, R. F. White. *Part I.:* S. Datta, T. H. A. Pinniger.

D.P.H.—J. Wesley Gilbert.

CONJOINT EXAMINING BOARD OF LONDON.—*Medicine:* G. A. Pennant. *Midwifery:* B. A. Astley-Weston, A. E. Young, H. E. Archer.

**University of Bristol.**—POSTGRADUATE STUDIES (Clinical Section). Weekly Clinical Demonstrations at the Royal Infirmary and General Hospital alternately, on Thursdays, 2.30—3.30 p.m., during May, June, July, October, November, December, 1919.

A course of Clinical Demonstrations has been arranged for Medical Practitioners by the University. Each demonstration will be given by a "team" composed of a Physician, a Surgeon, a Pathologist or a Specialist, and any others who have co-operated in the diagnosis and treatment of the case concerned. It will be illustrated by apparatus, books, specimens, tests and methods.

The course will include subjects associated with recent researches, recent epidemic outbreaks and general diseases on which new observations have been made.

In order that the Practitioner may also have opportunities for personal study and practice, attendants on the course will have the further advantage of admission for Medical Instruction for one month to any or all the departments of the Clinical Institutions included in the University.

FEE.—Three Guineas for the Course and Clinical work.

DAILY POSTGRADUATE STUDY.—For those who are able to devote several hours each day to Hospital practice, the University offers special facilities for Postgraduate work. Qualified Medical Practitioners may be appointed as *Clinical Assistants* for a period of one or more months. They may act as Assistants, if times permit, in more than one Department and in any of the Hospitals during their period of study. They will be entitled to the use of the Clinical Laboratories and Medical Library, and have the right to attend in all departments, including Operations, Postgraduate and ordinary Clinical Demonstrations and Post-mortem examinations.

FEE.—Three Guineas per month.

All inquiries and applications for admission should be addressed to the Director of Postgraduate Studies (Clinical Section), Pathological Department, University of Bristol.

The undermentioned are included in the Birthday Honours, and to them we offer our congratulations :—

C.B.E.—Lieut.-Col. and Brevet-Col. J. Paul Bush, C.M.G., Major (temp. Col.) J. Swain, C.B., Col. P. G. Stock, C.B., Temp. Lieut.-Col. J. H. Parsons, Capt. T. S. Hele.

O.B.E.—Major R. C. Clarke, Capt. R. S. S. Statham, Surgeon Lieut. A. E. Iles, Major (a/Lieut.-Col.) T. M. Carter, Capt. C. W. Wanklyn-James, Capt. C. C. Chesterman.

D.S.O.—Capt. (a/Lieut.-Col.) I. R. Hudleston, Capt. and Brevet-Major (a/Lieut.-Col.) P. S. Tomlinson.

### Bristol Medico-Chirurgical Society.

### President.

R. G. P. LANSDOWN, M.B., B.S.Durh.

### President-Elect.

I. WALKER HALL, M.D. Vict.

### Committee.

*Ex-Officio*
- FREDERICK LACE (Retiring President).
- P. WATSON-WILLIAMS, M.D. Lond. (Editor of *Journal*).
- C. K. RUDGE (Hon. Librarian).
- J. M. FORTESCUE-BRICKDALE, M.A., M.D., B.Ch.Oxon. (Editorial Secretary).

*Elected.*

GEORGE PARKER, M A., M.D. Cantab. } Ex-Presidents.
R. SHINGLETON SMITH, M.D., B.Sc. Lond. } Ex-Presidents.
J. LACY FIRTH, M.D., M.S. Lond.
J. O. SYMES, M.D. Lond.
L. E. V. EVERY-CLAYTON, M.D., B.S. Lond.
W. A. SMITH, M.A., M.B.
THOS. CARWARDINE, M.S.
D. C. RAYNER.

### Honorary Secretary.

A. J. WRIGHT, M.B., B.S. Lond.

Medical Practitioners wishing to join the Society are requested to communicate with the Hon. Secretary, Mr. A. J. WRIGHT, 14 Victoria Square, Clifton. The Annual Subscription is 21/-.

---

*Extracts from Journal By-laws.*

4.—The *Journal* shall be delivered free to Subscribers and also to Members of the Society whose subscriptions are not in arrear.

6.—The Annual Subscription to the *Journal* for those who pay before the 1st of July shall be 5/-, post free, or 6/- if paid after that date.

---

Communications intended for insertion in the *Journal* should be sent to the Editor, Dr. WATSON-WILLIAMS, 2 Rodney Place, Clifton, Bristol.

Books for Review and Exchange Journals should be sent to the Assistant-Editor, *Bristol Medico-Chirurgical Journal*, Medical Library, The University, Bristol.

Communications referring to the delivery of the *Journal* should be sent to the Editorial Secretary, J. M. Fortescue-Brickdale, 52 Pembroke Road, Clifton, Bristol, to whom Subscriptions are to be paid *in advance.*

Cases for Binding Vols. I—XXXV are now ready, and may be obtained, price 1/6 each (postage extra), upon application to J. W. ARROWSMITH LTD., Quay Street, Bristol; or the *Journal* will be bound, including Case, for 3/- per volume.

Owing to the war, no March or September issue of the 1916 *Journal* was published, so that Vol. XXXIV consisted of the two special parts numbered 130, 131, and only three parts were issued in 1917, so that Vol. XXXV consists of numbers 132, 133 and 134. One part was issued in 1918, Vol. XXXVI, number 135; this volume will be continued during 1919.

### Bristol Medico-Chirurgical Society.

### PAST PRESIDENTS.

Library of Congress SEP 13 1919 Smithsonian Deposit

1874—75. FREDERICK BRITTAN, M.D., B.A. Dub.
1875—76. ROBERT WILLIAM COE.
1876—77. SAMUEL MARTYN, M.D. Ed.
1877—78. AUGUSTIN PRICHARD, M.D. Berlin.
1878—79. HENRY EDWARD FRIPP, M.D. Würzburg.
1879—80. WILLIAM MICHELL CLARKE.
1880—81. JOSEPH GRIFFITHS SWAYNE, M.D. Lond.
1881—82. EDWARD LONG FOX, M.D. Oxon.
1882—83. JAMES GEORGE DAVEY, M.D. St. And.
1883—84. WILLIAM JOHNSTONE FYFFE, M.D., B.A. Dub.
1884—85. GEORGE FORSTER BURDER, M.D. Aberd.
1885—86. WILLIAM HENRY SPENCER, M.D., M.A. Cantab.
1886—87. FRANCIS POOLE LANSDOWN.
1887—88. LEMUEL MATTHEWS GRIFFITHS.
1888—89. ROBERT SHINGLETON SMITH, M.D., B.Sc. Lond.
1889—90. NELSON CONGREVE DOBSON, Ch.M. Bristol.
1890—91. SAMUEL HENRY SWAYNE.
1891—92. FRANCIS RICHARDSON CROSS, M.B. Lond., LL.D. Bristol.
1892—93. EDWARD MARKHAM SKERRITT, M.D., B.S., B.A. Lond.
1893—94. JAMES GREIG SMITH, M.B., C.M., M.A. Aberd., F.R.S.E.
1894—95. ALFRED JAMES HARRISON, M.B. Lond.
1895—96. ARTHUR WILLIAM PRICHARD.
1896—97. ALFRED EDWARD AUST LAWRENCE, M.D., C.M. Aberd.
1897—98. JOHN EDWARD SHAW, M.B., C.M. Ed.
1898—99. ROBERT ROXBURGH, M.B. Ed.
1899—00. WILLIAM HENRY HARSANT.
1900—01. DAVID SAMUEL DAVIES, M.D. Lond.
1901—02. BARCLAY JOSIAH BARON, M.B., C.M. Ed.
1902—03. GEORGE MUNRO SMITH, M.D. Bristol.
1903—04. JAMES PAUL BUSH, C.M.G., C.B.E., Ch.M. Bristol.
1904—05. REGINALD EAGER, M.D. Lond.
1905—06. JOHN DACRE.
1906—07. JAMES TAYLOR.
1907—08. HENRY WALDO, M.D., C.M. Aberd.
1908—09. JOHN MICHELL CLARKE, M.D., M.A. Cantab.
1909—10. JAMES SWAIN, C.B., C.B.E., M.D., M.S. Lond.
1910—11. BERTRAM MITFORD HERON ROGERS, M.D., B.Ch., B.A. Oxon.
1911—12. CHARLES ALEXANDER MORTON.
1912—13. WALTER CARLESS SWAYNE, M.D., B.S. Lond. and Bristol.
1913—14. PATRICK WATSON-WILLIAMS, M.D. Lond.
1914—15. WILLIAM HARRY CHRISTOPHER NEWNHAM, M.A., M.B. Cantab.
1915—16. GEORGE PARKER, M.A., M.D. Cantab.
1916—17. FRANCIS HENRY EDGEWORTH, M.A., M.D. Cantab. D.Sc. Lond.
1917—18. FREDERICK LACE.

1919.

---

LIST OF SUBSCRIBERS

TO THE

### Bristol Medico-Chirurgical Journal.

---

### HONORARY MEMBERS OF THE SOCIETY.

| | |
|---|---|
| Owen, Sir Isambard, D.C.L., M.D. | University of Bristol. |
| Dobson, N. C., Ch.M., F.R.C.S. ... | 16 College Road, Clifton. |
| Griffiths, L. M., M.R.C.S. ... ... | 11 Pembroke Road, Clifton. |
| Smith, R. Shingleton, M.D., B.Sc., F.R.C.P. ... ... ... ... ... ... | Deepholm, Clifton Park, Clifton. |

---

### SUBSCRIBERS.

*Those marked * are Members of the Society.*

| | |
|---|---|
| Ackland, J. McKno ... ... ... ... ... ... | 1 Barnfield Crescent, Exeter. |
| *Ackland, W. R., M.D.S. Bristol ... ... ... | 5 Rodney Place, Clifton. |
| Adye, W. J. A. ... ... ... ... .. ... ... ... | St. Margarets, Bradford-on Avon, Wilts. |
| Aldous, George F. ... ... ... ... ... ... ... | Charlton House, Mannamead, Plymouth. |
| *Alexander, D.A., M.B., Ch.B. Vict.... ... ... | 30 Berkeley Square, Clifton. |
| Alford, H. J., M.D. Lond. ... ... ... ... ... | 19 Park Street, Taunton. |
| *Askins, R.A., M.D., B.Ch., B.A.O. Dubl. ... | Guildhall, Bristol. |
| *Aubrey, T., M.B. Lond. ... ... ... ... ... | Bitton, near Bristol. |
| Aveline, H. T. S., M.D. Durh. ... ... ... ... | Somerset Asylum, Taunton. |
| *Baker, Lily A., M.D., B.Ch., B.A.O., R.U.I.... | 8 Richmond Hill, Clifton. |
| *Ball, E. J., M.A., Ph.D., Assoc. R.S. Mines, London ... ... ... ... ... ... ... ... ... | 120 Pembroke Road, Bristol. |
| *Ballance, H. Stanley M.D. Lond. ... ... | 1 Royal Terrace, Weston-super-Mare. |

### LIST OF SUBSCRIBERS.

| | |
|---|---|
| BARBER, M. C., M.B., Ch.B. Bristol ... ... | Ingledene. High Street, Staple Hill, Bristol. |
| *BARKER, G. H., M.D., B.Sc. Lond. ... ... ... | 124 Redland Road, Bristol |
| *BENSON, J. R. ... ... ... ... ... ... ... ... | Fiddington House, Market Lavington, Wilts. |
| *BERGIN, F. G. ... ... ... ... ... ... ... ... | 31 Oakfield Road, Clifton. |
| BERNARD, C. ... ... ... ... ... ... ... ... | 704 Fishponds Road, Fishponds, Bristol. |
| BERRY, F. C., M.D., B.Ch., B.A. Dub. ... ... | Locarno, Burnham, Somerset. |
| BERRY, JAMES, M.B., B.S. Lond. ... ... ... | 21 Wimpole Street, London, W.1. |
| BIAMONTI, Signor SEBASTIANO ... ... ... ... | 27 Via Balbi, Genoa, Italy. |
| *BLACKER, A. E.... ... ... ... ... ... ... ... | 20 Victoria Square, Clifton. |
| BLACKETT, J. F., M.D., B.S. Lond. ... ... ... | 10 South Stoke Road, Combe Down, Bath. |
| *BLAGG, ARTHUR F., M.D. Durh.... ... ... ... | 28 Caledonia Place, Clifton. |
| BODMAN, J. HERVEY, M.D. Lond. ... ... ... | 9 Whiteladies Road, Clifton. |
| *BOWKER, G. E., M.D. Edin.... ... ... ... ... | 11 North Parade, Bath. |
| *BRASHER, C. W. J., M.B., Ch.B. Bristol ... ... | 17 Oakfield Road, Clifton. |
| *BRISTOWE, H. C., M.D. Lond. ... ... ... ... | Wrington, Somerset. |
| *BROWN, WILLIAM, M.D., C.M. Glasg. ... ... | Park View, Fishponds, Bristol. |
| *BURDON-COOPER, J., M.D., B.Sc. Durh. ... ... | 12 The Circus, Bath. |
| BURROUGHS, E. F. H. ... ... ... ... ... ... | 36 Cornwallis Crescent, Clifton. |

| | |
|---|---|
| *CADMAN, E. S. R., M.D., C.M. Edin. ... ... | Gordon House, Westham, Pevensey, Sussex. |
| *CAIRNS, F. J. ... ... ... ... ... ... ... ... | Devon House, Whitehall Road, Bristol. |
| *CARLETON, H. H., Capt., M.A., M.D., B.Ch. (Oxon.) .. ... ... ... ... ... ... ... ... | Special Military Hospital, Southmead. |
| *CARLING, A., M.A., M.B., B.C. Cantab. ... ... | 12 Great George Street, Bristol. |
| *CARTER, T. M., M.D. Durh. ... ... ... ... | Lieut.-Col., R.A.M.C. |
| *CARWARDINE, THOMAS, M.B., M.S. Lond. ... | 16 Victoria Square, Clifton. |
| *CAVE, EDWARD J., M.D. Lond. ... ... ... ... | 20 Circus, Bath. |
| *CHARLES, J. R., M.A., M.D., B.C. Cantab. ... | 12 Victoria Square, Clifton. |
| *CHITTY, H., M.S. Lond. ... ... ... ... ... | 46 Pembroke Road, Clifton. |
| *CHRISTOFFERSON, P. E., M.B., Ch.B. Bristol .. | 8 Lansdown Place, Clifton. |
| CHUTE, HENRY M. ... ... ... ... ... ... ... | King William's Town, S. Africa. |
| *CLARKE, C., M.B., B.S. Lond. ... ... ... ... | 17 Elmdale Road, Clifton. |
| *CLARKE, R. C., O.B.E., M.B., Ch.B. Bristol ... | Amherst House, Clifton Park, Clifton. |
| CLOWES, E. F.... ... ... ... ... ... ... ... | 6 Trinity Street, Colchester. |
| CONNELLAN, Lieut. P. S., I.M.S. ... ... ... | c/o Grindlay & Co., 54 Parliament Street London, S.W. |
| *COOMBS, CAREY, M.D. Lond. ... ... ... ... | 3 Pembroke Road, Clifton. |
| *COOPER, WILLIAM RUSSELL ... ... ... ... | 13 Westfield Park, Redland, Bristol. |
| *CORFIELD, C. .. ... ... ... ... ... ... ... | 12 Pembroke Road, Clifton. |
| COSSHAM, W. R., M.D., C.M. Aberd.... ... ... | Wellesley House, Cirencester. |
| COTTON, WILLIAM, M.D., C.M., M.A. Ed. ... | 231 Gloucester Road, Bristol. |
| COULTER, R. J., M.B. Dub. ... ... ... ... ... | Ferncliffe, Stow Park Crescent, Newport, Mon. |
| CRIDLAND, A. B. ... ... ... ... ... ... ... | Salisbury House, Chapel Ash, Wolverhampton. |
| *CROSS, F. R., M.B. Lond., LL.D. Bristol ... | Worcester House, Clifton. |
| *CROSSMAN, F. W., M.B.... ... ... ... ... ... | White's Hill, Hambrook, near Bristol. |
| *CROUCH, C. PERCIVAL, M.B. Lond. ... ... ... | Villa Rosa, Weston-super-Mare. |

### LIST OF SUBSCRIBERS.

| | |
|---|---|
| *DACRE, JOHN ... ... ... ... ... ... ... ... | 14 Eaton Crescent, Clifton. |
| *DAVIES, D. S., M.D. Lond., D.P.H. Cantab. ... | 6 Lansdown Place, Clifton. |
| DENING, EDWIN ... ... ... ... ... ... ... | Manor House, Stow-on-the-Wold, Glos. |
| DEVINE, H., M.D., Lond. ... ... ... ... ... | Corporation Mental Hospital, Portsmouth. |
| *DIXON, T. B. ... ... ... ... ... ... ... ... | 134 Coronation Road, Bristol. |
| *DRAPES, T. L., M.B., B.C., B.A. Cantab. ... | St. Maur, Beaufort Square, Chepstow. |
| DUNBAR, ELIZA L. WALKER, M.D. Zürich ... | 9 Oakfield Road, Clifton. |
| DYMOKE, F. ... ... ... ... ... ... ... ... | 21 Cotham Road, Bristol. |
| *EDGEWORTH, F. H., M.D., B.C., M.A. Cantab., D.Sc. Lond. ... ... ... ... ... ... ... ... | 4 Richmond Park Road, Clifton. |
| EDWARDS, C. D., B.A., M.D. Cantab. ... ... | The Corderies, Chalford, Glos. |
| ELLIOT BROS. LTD. ... ... ... ... ... ... ... | *Australian Pharmaceutical Notes & News*, O'Connell Street, Sydney, N.S.W. |
| *ELLIOTT, CHRISTOPHER, M.D., B.A. Dub. ... | 102 Pembroke Road, Clifton. |
| ELLIOTT, F. PERCY, M.B., C.M. Aberd. ... ... | 113 Grove Rd., Walthamstow, London, E.17. |
| *EVERY-CLAYTON, L. E. V., M.D. Lond ... ... | Emsworth House, Clevedon, Somerset. |
| *FAILL, C. J. C. ... ... ... ... ... ... ... ... | 40 Princes Street, Bristol. |
| FARNFIELD, W. W. ... ... ... ... ... ... ... | Clive Vale, Gillingham, Dorset. |
| *FELLS, A., M.B., C.M. Edin. ... ... ... ... | 10 Elton Road, Clifton. |
| *FIRTH, J. L., M.D., M.S. Lond. ... ... ... ... | 8 Victoria Square, Clifton. |
| *FLEMMING, A. L., M.B., Ch.B. Bristol ... ... | 48 Pembroke Road, Clifton. |
| *FLEMMING C. E. S. ... ... ... ... ... ... | Manvers House, Bradford-on-Avon. |
| *FORTESCUE-BRICKDALE, J. M., M.A., M.D., B.Ch. Oxon. ... ... ... ... ... ... ... | 52 Pembroke Road, Clifton. |
| FOSS, E. V., M.D., Ch.B. Bristol ... ... ... | Clouds Hill House, St. George, Bristol. |
| *FRASER, FORBES ... ... ... ... ... ... ... | 5 The Circus, Bath. |
| FREEMAN, J., M.D. Brux. ... ... ... ... ... | Waterloo Villa, 105 Queen's Road, Clifton. |
| GARLAND, E. B., M.B., C.M. Edin. ... ... ... | 114*a* Hampton Road, Redland, Bristol. |
| *GERRISH, D. S., M.R.C.S. Lond.... ... ... ... | 328 & 330 Church Road, St. George, Bristol. |
| *GIBBS, A. N. GODBY ... ... ... ... ... ... | 52 Whiteladies Road, Clifton. |
| *GLENNY, G. T., M.B., B.S. (Lond.) ... ... ... | 102 Pembroke Road, Clifton. |
| *GRATTE, C. BROOKE ... ... ... ... ... ... | 18 Gold Tops, Newport, Mon. |
| *GREEN, T. A., M.D., C.M. Edin. ... ... ... | 33*a* Whiteladies Road, Clifton. |
| GREEN-ARMYTAGE, Capt. V. B., I.M.S. ... ... | c/o T. Cook & Son, Calcutta. |
| *GREER, W. J. ... ... ... ... ... ... ... ... | 19 Gold Tops, Newport, Mon. |
| GRIFFITHS, CORNELIUS A. ... ... ... ... ... | 35 Newport Road, Cardiff. |
| *GRIFFITHS, J. S. ... ... ... ... ... ... ... | 20 Redland Park, Bristol. |
| *GROVES, E. W. H., M.D., M.S. Lond. ... ... | 25 Victoria Square, Clifton. |

### LIST OF SUBSCRIBERS.

| | |
|---|---|
| *Hailes, Clements D. G., M.D., C.M. Ed. ... | 2 Richmond Park Road. |
| *Hall, E. G., M.B. Lond. ... ... ... ... ... ... | 42 Apsley Road, Clifton, Bristol. |
| *Hall, I. Walker, M.D., Ch.B.Vict. ... ... | Linden Gate, Clifton Down Road, Clifton. |
| Handfield-Jones, Montagu, M.D. Lond. ... | 36 Cavendish Square, London, W.1. |
| *Harris, H. Elwin, B.A., M.B. Cantab. ... ... | 13 Lansdown Place, Clifton. |
| *Harsant, W. H. ... ... ... ... ... ... ... ... | Tower House, Clifton Down Road, Clifton. |
| *Harty, J. P. I., M.B., B.Ch., B.A.O., R.U.I. | 7 Percival Road, Clifton. |
| Hawes, C. S. ... ... ... ... ... ... ... ... ... | 8 Elsworthy Road, London, N.W.3. |
| Hayman, Howard ... ... ... ... .. ... ... | 3 Clifton Park, Clifton. |
| Hele, T. S., M.A., M.B. ... ... ... ... ... | Emmanuel College, Cambridge. |
| *Herapath, C. E. K., M.D. Lond. ... ... ... | 12 Brunswick Square, Bristol. |
| *Herapath, C. K. C. ... ... ... ... ... ... ... | Stoneleigh, Cotham Park, Bristol. |
| Hill, Hedley, M.D. Durh. ... ... ... ... | 1 Whiteladies Road, Clifton. |

Hospital, Bristol General (*Secretary*, T. W. Gregg).

| | |
|---|---|
| *Iles, A. E., O.B.E., M.B., B.S. Lond. ... ... | 19 Belgrave Rd., Tyndall's Park, Bristol. |

Infirmary, Bristol Royal (*Secretary*, W. E. Budgett).

| | |
|---|---|
| Ingle, C. Durban ... ... ... ... ... ... ... ... | Somerton, Somerset. |

| | |
|---|---|
| *James, C. W. W. ... ... ... ... ... ... ... ... | Trevena, Highbury Hill, Weston-super-Mare. |
| Jamison, A., M.B., B.Ch., M.A. R.U.I. ... ... | Ardenvohu Woodstock Road, Belfast. |
| *Joll, C. A., M.B., M.S. Lond. ... ... ... ... | 21 Wimpole St., Cavendish Sq., W.1. |
| *Jones, J. Ellington ... ... ... ... .. ... | 8 Chantry Road Clifton. |
| Joseph, A. Hill, M.D. Lond. ... ... ... ... | Bexhill-on-Sea. |

| | |
|---|---|
| *Kay-Mouat, J. R., M.A. Oxon., M.B., Ch.B. Bristol ... ... ... ... ... ... ... ... ... | University of Bristol. |
| *Kerfoot, Stanley J. ... ... ... ... ... ... | 109 East Street, Bedminster, Bristol. |
| *Kerr, R. A., M.B., B.Ch. ... ... ... ... ... | 40 Orients Gardens, Belfast, Ireland. |
| *Kyle, H. G., M.A., M.D. B.Ch. Oxon. ... ... | 31 Westbury Road, Bristol. |

| | |
|---|---|
| *Lace, F. ... ... ... ... ... ... ... ... ... ... | 5 Gay Street, Bath. |
| *Lansdown, R. G. P., M.D., B.S. Durh. ... ... | 39 Oakfield Road, Clifton. |
| *Lavington, C. C., M.B., B.S. Durh. ... ... | 1 Burlington Road, Redland, Bristol. |
| Lendon, A. A., M.D. Lond. ... ... ... ... | North Terrace, Adelaide, Australia. |
| *Lees, E. Leonard, M.D., C.M. Ed. ... ... ... | 1 Chesterfield Place, Clifton. |

### LIST OF SUBSCRIBERS.

| | |
|---|---|
| Leigh, W. W. | Treharris, Glamorganshire. |
| *Leonard, R. C., M.D. Durham | Coronation Road. Bristol. |
| *Linton, Marion S., M.B. Lond. | 21 Oakfield Road, Clifton. |
| *Llewellyn, R. Ll. J., M.B. Lond. | 41 Gay Street, Bath. |
| *Longley, J. A. Noel, M.B., Ch.B. Birm. | 124a Redland Road, Bristol. |
| *Lucas, J. J. S., M.D. Lond. | 186 Wells Road, Totterdown, Bristol. |

| | |
|---|---|
| Mackinnon, Charles, M.B., C.M., M.A. Glasg. | Davaar, Cirencester. |
| Maclean, Ewen J., M.D., C.M. Ed. | 12 Park Place, Cardiff. |
| Maclean, J. Campbell, M.B., C.M. Ed. | Swindon House, Swindon, Wiltshire. |
| *MacLeod, Donald, M.B., C.M. Ed. | 84 Redland Road, Redland, Bristol. |
| *MacWatters, J. C. | Almondsbury, near Bristol. |
| Marshall, Arthur L., M.B., B.A. Cantab. | Thornleigh, Upper Clapton, London, E.5. |
| *Martin, E. F., M.D., C.M. Ed. | 7 Royal Terrace, Weston-super-Mare. |
| *Martyn, G. J. King, M.D., B.C., B.A. Cantab. | 8 Gay Street, Bath. |
| Mason, S. B. | 12 Park Terrace, Pontypool. |
| Miall, C. L'O. | Elm Hayes, Paulton, near Bristol. |
| *Moore, C. A., M.B., M.S. Lond. | 56 St. Paul's Road, Clifton. |
| Morris, L. N., M.B., C.h.B. Bris. | 24 All Hallows Road, Upper Easton, near Bristol. |
| *Morris, Mary E. H., M.D. Lond. | 19 Gay Street. Bath. |
| *Morton, C. A., F.R.C.S. Eng. | 14 Vyvyan Terrace, Clifton. |
| Munden, Charles | Ilminster, Somerset. |
| *Munro, J. M. H., D.Sc. Lond. | 12 Grosvenor Place, Bath. |
| *Myles, G T. | 7 Apsley Road, Clifton. |

| | |
|---|---|
| *Neild, Newman, M.B., B.Ch. Vict. | 9 Richmond Hill, Clifton. |
| *Newnham, W. H. C., M.B., M.A. Cantab. | 3 Lansdown Place, Victoria Square, Clifton. |
| *Nichols, F. C., M.B., Ch.B. Bristol | 38 Whiteladies Road, Clifton. |
| *Nixon, J. A., C.M.B., B.A., M.D., B.C. Cantab. | 7 Lansdown Place, Clifton. |

| | |
|---|---|
| Oppenheimer, Son & Co. Ltd. | 179 Queen Victoria Street, London, E.C.4. |
| *Ormerod, H. Lawrence, M.B., B.Ch. R.U.I. | 2 Henleaze Road, Westbury Park, Bristol. |
| *Orr-Ewing, H. J., M.B., B.S. Lond. | Halmer, South Road, Weston-snper-Mare. |

| | |
|---|---|
| Page, G. Shepley | 78 Old Market Street, Bristol. |
| *Parker, George, M.D., M.A. Cantab. | 14 Pembroke Road, Clifton. |
| Parkinson, Capt. G. S., R.A.M.C. | |
| *Parsons, H. F. | The Heath, Hazelwood Road, Sneyd Park. |

### LIST OF SUBSCRIBERS.

Parsons, J. H., M.B., B.S., D.Sc. Lond.... ... 54 Queen Anne Street, London, W.
Paterson, Donald Rose, M.D., C.M. Ed. ... 15 St. Andrew's Crescent, Cardiff.
Phillips, C. M., M.D. Brux. ... ... ... ... 502 Bath Road.
*Pickering, C. F. ... ... ... ... ... ... ... 13 Berkeley Square, Bristol.
*Pollard, J., M.B., B.Ch., B.A.O. ... ... ... 52 Cumberland Road, Bristol.
Powell, J. J., M.D. Lond. ... ... ... ... ... 2 Gloucester Road, Bishopston, Bristol.
Price, D. T. M.B. Lond. ... ... ... ... ... Castle Cary, Somerset.
*Prichard, Arthur W. ... ... ... ... ... ... 6 Rodney Place, Clifton.
*Prowse Arthur B., M.D. Lond. ... ... ... 5 Lansdown Place, Clifton.

*Rattray, J. M., M.D., C.M., M.A. Aberd. ... Rook Lane House, Frome, Somerset.
*Rayner, D. C., F.R.C.S. Eng. ... ... ... ... 9 Lansdown Place, Clifton, Bristol.
*Richardson, T. A. ... ... ... ... ... ... ... 10 Clifton Park Road, Clifton.
Ritchie, Thomas P. ... ... ... ... ... ... ... 27 Oakfield Road, Clifton.
*Robertson, D., M.B., Ch.B. Edin. ... ... ... 205 Wells Road, Knowle.
*Rogers, B. M. H., M.D., B.Ch., B.A. Oxon. ... 14 Mortimer Road, Clifton.
*Rolfe, R., B.A., M.B., B.C. Cantab. ... ... Park House, St. Andrews Road, Avonmouth.
*Rose, F. H. ... ... ... ... ... ... ... ... Westfield, Portishead, Somerset.
*Rossiter, George F., M.B. Lond. ... ... ... Cairo Lodge, Weston-super-Mare.
*Rudge, C. King ... ... ... ... ... ... ... 145 Whiteladies Road, Bristol.
*Rutherford, J. M., M.B., C.M. Edin., F.R.C.P. Brislington House, near Bristol.

Seward, W. J., M.B. Lond. ... ... ... ... 15 Chandos Avenue, Oakleigh Park, London, N.
*Short, A. R., M.D., B.S., B.Sc. Lond. ... ... Montrose House, Queen's Road, Clifton
*Short, L. J., M.D., B.S. Lond., M.D. Bris. ... Eton Villa, The Shrubbery, Weston-super-Mare.
*Smith, G. Cockburn, M.D. Brux. ... ... ... 14 South Road, Newton Abbot.
Smith, L. Shingleton, B.A., M.B. Cantab.... Camden Road, Brecon.
Smith, Rev. Reginald, M.A. Cantab. ... ... The Vicarage, Walpole St. Andrew Norfolk.
Smith, W. Steele, M.B. Lond. ... ... ... Caldecote, Cambridge.
*Smith, W. Alexander, M.B., M.A. Oxon. ... 70 Pembroke Road, Clifton.
Society, Cardiff Medical (*Hon. Lib.*, Queen Street, Cardiff).
Society, Manchester Medical ... ... ... Medical Society's Library, The Owens College, Manchester.
*Stack E. H. E., B.A., M.B., B.C. Cantab. ... Arvalee, Clifton Down Road, Clifton.
Staple, J. D. ... ... ... ... ... ... ... ... St. Martin's, Lyme Regis, Dorset.
*Statham, R. S. S., O.B.E., M.D. Bristol... ... 5 Westbourne Place, Clifton.
*Stock, W. S. V., M.B., B.S. Lond. ... ... ... 1 Mortimer Road, Clifton.
*Swain, James, M.D., M.S. Lond., C.B. ... ... 4 Victoria Square, Clifton.
*Swayne, W. Carless, M.D. Lond. ... ... ... 2 Clifton Park, Clifton.
*Symes, J. O., M.D. Lond. ... ... ... ... ... 71 Pembroke Road, Clifton.
Symonds, H. P. ... ... ... ... ... ... ... 35 Banbury Road, Oxford.

| | |
|---|---|
| TAYLOR, A. L. | The Royal Infirmary, Bristol. |
| TAYLOR, H. N., M.A. Cantab., M.D. Dublin | Hillside, Ebbw Vale. |
| *TAYLOR, JAMES | 36 Alma Road, Clifton |
| *TAYLOR, J. W., M.D. Bristol | 8 Redcliff Parade West, Bristol. |
| *TEMPLE, G. H., M.B., C.M. Ed. | Ailanthus, Bristol Road, Weston-super-Mare. |
| THIN, JAMES | 54 & 55 South Bridge, Edinburgh. |
| *THOMAS, J. D., M.B. Cantab. | Northwoods, Winterbourne. |
| THOMAS, J. LYNN, C.B. | Green Lawn, Pen-y-lan, Cardiff. |
| *THOMAS, J. TUBB, D.P.H. | The Halve House, Trowbridge. |
| *THOMAS, R. E., M.D., B.S. Lond. | Hospital and Sanatorium, Ham Green, Pill. |
| *THOMSON, F. G., M.A., M.D. Lond. | 20 Gay Street, Bath. |
| TOSSWILL, LEONARD R., D.P.H. Oxon. | 13 Tufnell Park Road, London, N.7. |

| | |
|---|---|
| VACHELL, HERBERT R., M.D., C.M. Aberd. | 18 Newport Road, Cardiff. |
| *VICKERY, W. H. | 1 Trewartha Park, Weston-super-Mare. |

| | |
|---|---|
| *WALDO, HENRY, M.D., C.M. Aberd. | 19 Pembroke Road, Clifton. |
| *WALKER, CYRIL H., M.B., B.A. Cantab. | 8 Oakfield Road, Clifton. |
| *WALLACE, JOHN | Lauriston, 56 Knightstone Road, Weston-super-Mare. |
| WALLACE, J. T., M.B., B.Ch., B.A. R.U.I. | 48 Coronation Road, Bedminster, Bristol. |
| *WALTERS, C. F. | 5 Mortimer Road, Clifton. |
| *WATERHOUSE, R., M.D. Lond. | 25 Circus, Bath. |
| *WATSON-WILLIAMS, P., M.D. Lond. | 2 Rodney Place, Clifton. |
| *WHICHER, A. H. | 115 Queen's Road, Clifton. |
| WHITE, J. W. | Orchard House, Nailsea. |
| *WHITE, PERCY W. | 37 Whiteladies Road, Clifton. |
| WIGAN, C. L., M.B., B.S. Durham | Clarence House, Portishead. |
| WIGMORE, J., M.D. R. U. I. | 16 Queen Square, Bath. |
| WILLCOX, R. LEWIS | 97 London Road, Salisbury, Wilts. |
| WILLETT, GEORGE G. D. | Keynsham, Somerset. |
| *WILLIAMS, E. C., M.B., B.C., B.A. Cantab. | 159 Whiteladies Road, Clifton. |
| WILLIAMS, H. EGERTON | The Mount, Newport, Mon. |
| WILLIAMS, S. R., M.B. Lond. | Buckfastleigh, Devon. |
| *WILLIAMSON, G. SCOTT | The General Hospital, Bristol. |
| *WILLS, W. K., M.B., B.C. Cantab. | 19 Whiteladies Road, Clifton. |
| *WINDSOR-AUBREY, H. W. | 79 Coronation Road, Bristol. |
| *WOOD, DUNCAN | The Porch, Corsham, Wilts. |
| *WOOD, W. V. | Langford, near Bristol. |
| *WRIGHT, A. J., M.B., B.S. Lond. | 14 Victoria Square, Clifton. |

| | |
|---|---|
| YOUNG, J., M.D. Glas. | |

### PUBLICATIONS RECEIVED.

From Félix Alcan, Paris:

*Pathologie de Guerre du Larynx et de la Trachée.* Par E. J. Moure, G. Liébert et G. Canuyt. 1918. Price 26 fr. 5 c.

From Baillière, Tindall and Cox, London:

*The "Nauheim" Treatment, in England, of Diseases of the Heart and Circulation.* By Leslie Thorne Thorne, M.D., B.S. Fifth Edition, 1918.

*The Treatment of Infected Wounds.* By A. Carrel and G. Dehelly. Translation by H. Child. Second Edition, 1918. 6s. net.

From John Bale, Sons and Danielsson, Ltd., London:

*Hæmatologist's Aid to Memory.* By Henry Harold Scott, M.D. 5s. net.

From Cassell and Company, Ltd., London:

*Surgical Applied Anatomy.* By Sir Frederick Treves, Bart., G.C.V.O., C.B., LL.D., F.R.C.S. Seventh Edition, revised by Arthur Keith, M.D., LL.D., F.R.C.S., F.R.S., and W. Colin Mackenzie, M.D., F.R.C.S. 1918. 10s. 6d. net.

*The Nation's Welfare: The Future of the Medical Profession.* By Major-General Sir Bertrand Dawson, G.C.V.O., C.B., M.D. 1918. Price 6d.

*Elements of Surgical Diagnosis.* By Sir Alfred Pearce Gould, K.C.V.O., M.S., F.R.C.S., and Eric Pearce Gould, M.D., M.Ch., F.R.C.S. Fifth Edition, revised. 1919, 12s. 6d. net.

From The Director, British Museum (Natural History), London:

*Map showing Distribution in England and Wales of the Anopheline Mosquitoes, with Explanatory Text.* By William Dickson Lang, M.A. 1918. Price 2s. 6d.

From Henry Frowde and Hodder & Stoughton, London:

*Scopolamine-Morphine.* By Wm. Osborne Greenwood, M.D., B.S. 1918. Price 6s. net.

*Burns and their Treatment.* By J. M. H. Macleod, M.A., M.D. 1918. Price 6s. net.

*Military Surgery.* By Dunlop Pearce Penhallow, S.B., M.D. With Introduction by Sir Alfred Keogh, K.C.B. Second Edition. 1918. Price 21s. net.

*The Diagnosis and Treatment of Venereal Diseases in General Practice.* By L. W. Harrison, D.S.O. Price 21s. net.

*Applied Bacteriology. Studies and Reviews of Some Present-day Problems.* Edited by C. H. Browning, M.D., D.P.H. 1918. Price 7s. 6d. net.

*Diseases of the Heart.* By Frederick W. Price, M.D. 1918. Price 21s. net.

*Bipp Treatment of War Wounds.* By Rutherford Morison. 1918. 2s. 6d. net.

*The Prevention of Venereal Diseases.* By Otto May, M.D. 1918. 7s. 6d. net.

*Amputation Stumps: Their Care and After Treatment.* By G. Martin Huggins, F.R.C.S. 1918. 7s. 6d. net.

*Vaccines and Sera.* By A. Geoffrey Shera, B.A., M.D., B.C. With an Introduction by Sir Clifford Allbutt, K.C.B., M.D., F.R.S. 1918. Price 7s. 6d. net.

*The Hearts of Man.* By A. M. Wilson, M.B. 1918. Price 6s. net.

*Gymnastic Treatment for Joint and Muscle Disabilities.* By Brevet-Colonel H. E. Deane, R.A.M.C. 1918. Price 5s. net.

*The Operative Treatment of Chronic Intestinal Stasis.* By Sir W. Arbuthnot Lane, Bart, C.B. Fourth Edition. 1918. 20s. net.

*Manual of Bacteriology.* By Robert Muir, M.A., M.D., and James Ritchie, M.A., M.D. Seventh Edition. 1919. Price 16s. net.

*A Manual of Elementary Zoology.* By L. A. Borradaile, M.A. Second Edition. 1918. Price 16s. net.

*Trench Fever. Report of Commission, Medical Research Committee.* American Red Cross. 1918. Price 21s. net.

*Orthopædic Effects of Gunshot Wounds and their Treatment.* By S. W. Daw, M.B., B.S. F.R.C.S. 1919. Price 7s. 6d. net.

*Surgical Aspects of Typhoid and Para-Typhoid Fevers.* By A. E. Webb-Johnson, D.S.O., M.B., F.R.C.S. With Foreword by Lieut.-General T. H. Goodwin, C.B., C.M.G., D.S.O. 1919. Price 10s. 6d. net.

*A Medical Field Service Handbook.* By C. Max Page, D.S.O., M.S., F.R.C.S. With Foreword by Major-General Sir George Makins, G.C.M.G., C.B. 1919. Price 6s. net.

*The Anatomy of Peripheral Nerves.* By A. Melville Paterson, M.D., F.R.C.S. 1919. Price 12s. 6d. net.

*The Forms of Alcoholism and their Treatment.* By Hugh Wingfield, M.A., M.D., B.C. 1919. Price 5s. net.

From H. K. Lewis and Co., Ltd., London:

*Glaucoma.* By Robert Henry Elliot. 1918. 21s. net.

*The Action of Muscles.* By William Colin MacKenzie, M.D., F.R.C.S. 1918. Price 12s. 6d. net.

### PUBLICATIONS RECEIVED (*continued*).

From Longmans, Green and Co., London :
*Principles of General Physiology.* By William Maddock Bayliss, M.A., D.Sc., F.R.S. Second Edition, revised. 1918. Price 24s. net.
*Anatomy, Descriptive and Applied.* By Henry Gray, F.R.S., F.R.C.S. Twentieth Edition. Edited by Robert Howden, M.A., D.Sc., M.B., C.M. 1918. Price 37s. 6d. net.

From Macmillan and Co., Limited London :
*Hysterical Disorders of Warfare.* By Lewis R. Yealland, M.D. With Preface by E. Farquhar Buzzard, M.D., F.R.C.P. 1918. Price 7s. 6d. net.
*The Soldier's First Aid.* By R. C. Wood, Q.M.S., Army Medical Corps. 1917. Price 2s. 6d. net.
*Typhoid Fever.* By Frederick G. Gay. 1918. Price 12s. 6d. net.

From Humphrey Milford, Oxford University Press, London :
*Seale-Hayne Neurological Studies.* Volume I., No. 1, July, 1918. Price 3s. 6d. net.

From Dr. D'Arcy Power, London :
*The Bradshaw Lecture delivered before the Royal College of Surgeons of England, November 14th,* 1918. By D'Arcy Power, M.B., F.R.C.S. 1919. [Reprint.]

From The School Medical Officer, Bristol Education Committee, Bristol :
*Annual Report of the Acting School Medical Officer.* 1917.

From John Wright and Sons, Ltd., Bristol :
*The Fitting Out and Administration of a Naval Hospital Ship.* By Edward Sutton Fleet Surgeon, R.N. 1918.
*War Wounds of the Lung.* By Pierre Duval. Authorised English Translation. 1918. Price 8s. 6d. net.
*An Index of Prognosis and End-Results of Treatment.* By Various Writers. Edited by A. Rendle Short, M.D., B.Sc., F.R.C.S. Second Edition, revised and enlarged. 1918. Price 30s. net.
*Our Baby.* By Mrs. J. Langton Hewer. Sixteenth Edition. 1918. 2s. 6d. net.
*Pye's Surgical Handicraft.* Eighth Edition. Edited and largely rewritten by W. H. Clayton-Greene, B.A., M.B., B.C., F.R.C.S. 1919. Price 21s. net.

---

### Bristol Medico-Chirurgical Journal

*Established in 1883 as a Journal of the Medical Sciences for the West of England and South Wales.*

### Terms for Advertisements.

| | £ | s. | d. |
|---|---|---|---|
| Whole Page ... ... ... ... | **1** | **1** | **0** |
| Half Page ... ... ... ... ... | **0** | **12** | **6** |
| Quarter Page ... ... ... ... | **0** | **7** | **6** |

Insets (2 or 4 pages), 20/-

Discount of 5 per cent. will be allowed if payment is made at the time of ordering.

Terms for special positions upon application.

*Orders to be sent to the Publishers—*

J. W. ARROWSMITH LTD., Quay Street, Bristol.

